# Health sector leadership to strengthen civil registration and vital statistics systems

**DOI:** 10.2471/BLT.22.289038

**Published:** 2023-12-01

**Authors:** Alan D Lopez

**Affiliations:** a LM Consulting, 40 Caryota Court, Tamborine Mountain, 4272 Queensland, Australia.

For governments to control outbreaks and address the complex demands of changing disease and injury patterns, they need dependable health intelligence. The cornerstone of this information base is a reliable and functioning civil registration and vital statistics system that provides governments with accurate and timely information on who is dying of which causes and at what ages, differentially in various population subgroups, and how these patterns are changing.

Understanding the levels and patterns of fertility in a population on a continuous and disaggregated basis is similarly important for planning maternal and child health care, as well as for schooling and other social benefits and programmes. Functioning civil registration and vital statistics systems serve this need, since information on birth registration derived from surveys is potentially biased and generally out of date.^1^

Good quality civil registration and vital statistics systems are also essential for reliably monitoring progress with national health and development goals, particularly the health-related sustainable development goals.^2^ To make informed decisions about the policies and programmes that are needed to optimize population health, increase health system efficiency and accelerate progress towards universal health coverage, governments depend upon the availability of recent and reliable birth and death data. Yet, poor quality and incomplete vital statistics have been repeatedly identified as the weakest component of national health information systems.^3^^,^^4^

As the primary provider of services around the time of birth – and increasingly around the time of death – the health sector is well placed to ensure that governments obtain the information they need on fertility and mortality. Recognizing this key yet underexploited interface between the health sector and civil registration systems, the World Health Organization (WHO) has developed a comprehensive *Civil registration and vital statistics strategic implementation plan 2021–2025*^5^ to stimulate, guide and support efforts by Member States to enhance the role of the health sector in developing these key data for development, including guidance for health managers on practical, operational steps that the health sector can take to improve birth and death registration.

A fundamental component of WHO’s implementation plan is to ensure a comprehensive and comparable understanding of the current status and functioning of civil registration and vital statistics systems in countries, to better target improvement efforts and monitor progress at national, regional and global levels. The three studies that I co-authored in this issue of the *Bulletin of the World Health Organization* provide scientific and comparable assessments of the availability, completeness and quality of birth, death and cause-of-death data worldwide, as well as of the operational characteristics of civil registration and vital statistics systems in countries. One article provides a detailed overview of the performance, strengths and weaknesses of civil registration and vital statistics systems in countries, based on a standardized and comparable assessment framework that synthesizes all available information from previous global assessments.^6^ The two other articles assess in more detail the availability and quality of vital statistics on births and deaths separately, focusing on indicators likely to be of greatest relevance for public policy.^7^^,^^8^ Their analyses provide a comprehensive account of the state of the world’s vital statistics.

The results are grim. Vital statistics, so fundamental for guiding health and social sector planning, do not exist for many countries, and where they do, are often of insufficient quality to reliably inform policy. Poor data consolidation, transmission and quality assessment practices limit their usefulness, while lack of analytical capacity in countries often means that the full policy potential of birth and death data is not being realized.

Given the major advances in population health measurement research over the past two decades,^9^ and the rapidly accumulating experience with the application of new data collection techniques, artificial intelligence and information technology applications,^10^^,^^11^ rapid progress to address these challenges and thus improve the policy utility of national vital registration systems is now feasible. Indeed, the health sector can and should provide national vital registration systems with more active and purposeful leadership to support automated notifications of births and deaths from health facilities, improve cause-of-death certification skills, and promote the routine incorporation of automated verbal autopsy methods to attribute cause for deaths at home. 

The three papers provide a timely and sobering view of the state of birth and death data worldwide and of the data systems that generate them. The large data gaps identified are unacceptable, given the critical role of reliable vital statistics in development strategies and promoting health for all. While the technology and partnerships to make rapid progress in creating reliable vital statistics exist,^10^^–^^12^ effective global health leadership is essential.
